# Developmental biology, the stem cell of biological disciplines

**DOI:** 10.1371/journal.pbio.2003691

**Published:** 2017-12-28

**Authors:** Scott F. Gilbert

**Affiliations:** Department of Biology, Swarthmore College, Swarthmore, Pennsylvania, United States of America

## Abstract

Developmental biology (including embryology) is proposed as "the stem cell of biological disciplines.” Genetics, cell biology, oncology, immunology, evolutionary mechanisms, neurobiology, and systems biology each has its ancestry in developmental biology. Moreover, developmental biology continues to roll on, budding off more disciplines, while retaining its own identity. While its descendant disciplines differentiate into sciences with a restricted set of paradigms, examples, and techniques, developmental biology remains vigorous, pluripotent, and relatively undifferentiated. In many disciplines, especially in evolutionary biology and oncology, the developmental perspective is being reasserted as an important research program.

We were finishing dinner at a conference on evolutionary developmental biology when a graduate student asked me to explain some comments I’d made during a question and answer session. I had disagreed with a colleague’s reliance on citation analysis to present a history of evolutionary developmental biology. Citation lists are political documents, I had argued. Citations don’t reveal whether a paper had influenced the author, or even whether the author had read it. Furthermore, a history of a new field should explain why the field arose. It might even have a mythos, a narrative theme for its origin story.

The student asked if my account of the history of evolutionary developmental biology had an underlying narrative and, if so, what it was. I told him something like, “Yes. If you analyze my accounts, you’ll find that there is an underlying narrative, and that narrative is ‘the return of the rightful sovereign.’ Development was originally seen as the motor of evolution, and the principal way of explaining evolution was through embryology. In fact, in the late 1800s, the word ‘evolution’ could mean either phylogenetic or embryological development. But genetics arose out of embryology, and eventually, evolution came to be seen as a proper subset of population genetics. Genetics displaced development as the way to study evolution. In my narrative, evo-devo represents the return of developmental biology to its rightful place as the means to study evolution.”

Sensing he didn’t get the connection, I continued. “The return of the rightful sovereign. Remember Errol Flynn’s *Robin Hood*, in which the captured monk dramatically sheds his clerical garb to reveal himself as King Richard, returned to England to correct John’s injustices?”

My dated allusion was not getting through, either. “*Game of Thrones*,” I hazarded.

“Yes!” he exclaimed, “I get it. Evo-Devo and *Game of Thrones*!”

As I recalled my version of developmental biology’s origin story, I pondered a larger question, which others had also noted [1]: Why and how has developmental biology, once a central focus of biology, been marginalized in our curriculum? Nobel Prizes and other awards for discoveries in developmental biology are often cast (even in scientific journals) as breakthroughs in genetics or in stem cell biology. Journal articles pertaining directly to developmental biology are often catalogued under “cancer biology,” “evolution,” or “neurobiology.” Developmental biology has even been disparaged as “old fashioned” by experts in the field who are doing it excellently, but who prefer to call it something else. In the most recent meeting of the Society for Developmental Biology, the president of the society, Blanche Capel [2], asked in her presidential address, “Did you ever think, like me, that Developmental Biology does not get the credit it deserves for its contributions to understanding the natural world?”

As should be clear by now, I have indeed wondered why developmental biology has been overlooked and am playing with a hypothesis to explain why. I propose that developmental biology (and its parent discipline, embryology) has been the stem cell of biological disciplines. It is not a “differentiated” discipline, but the pluripotent discipline that generates disciplines like genetics and immunology, all the while retaining its own identity.

(Developmental biology, it should be noted, is a twice-named discipline. In the 1950s, the term was coined by Paul Weiss and N. J. Berrill to include the parent discipline, embryology, as well as the study of adult stem cells and nonembryonic development, such as budding and regeneration. This was the impetus for the journal *Developmental Biology*. It was named again in the 1970s, for the annual series, *Current Topics in Developmental Biology*, where it was seen as the molecular approach to embryology. In both cases, “developmental biology” was viewed as the modernization and extension of embryology [3].)

So, let us begin with the cell theory. In the mid-1800s, the study of embryos gave rise to various theories of cell formation. Schleiden, Schwann, and Remak formed their cell theories to answer the question of how multicellular embryos emerged, and thus gave rise to the discipline of cytology/cell biology [4–6]. Today’s cell theory is largely based on the 1862 hypothesis of Robert Remak [7], who first figured out that the embryo is constructed by cell division and that all the cells of the body are descendants of the zygote. But where do these cells form? By the turn of that century, Eli Metchnikoff and other embryologists, looking for the sources and roles of the mesoderm (the middle cell layer of embryonic embryo), formulated the first approaches to immunology. Metchnikoff had found that the mesodermal cells of the starfish embryo budded off from the gut-producing endoderm and were capable of their own intracellular digestion, phagocytizing foreign bodies inserted into the larvae. His discovery led to the first hypotheses of cellular immunity [8]. Thus, by 1900, embryology had already given rise to cell biology and immunology.

Shortly thereafter, the gene theory was constructed by embryologists who had been embroiled in debates over what part of the embryo—the nucleus or the cytoplasm—controlled development. In the early 1900s, embryologists Theodor Boveri and E. B. Wilson believed that the nucleus, especially the nuclear chromosomes, carried the instructions for organismal development. In contrast, embryologist Thomas Hunt Morgan (who had written a monograph on the embryology of the frog egg) favored the cytoplasm [9]. By 1915, Morgan [10] inadvertently obtained the evidence that chromosomal genes were necessary for the production of inherited traits. (He had hoped to prove otherwise). Another embryologist, William Bateson, would later call this new field “genetics,” and Morgan [11] would formally separate the two fields, saying that genetics studied the transmission of inherited traits, whereas embryology studied their expression. While earlier genetics (the “assortment” phase) had been suggested by breeders such as Mendel, the field we now know as genetics (studying traits whose segregation and assortment can be explained by the locations of specific genes on particular chromosomes) came from the chromosomal studies of embryologists such as Morgan and Wilson, supplemented by the theoretical discussions and analyses of embryologist Theodor Boveri and Wilson’s graduate student, Walter Sutton.

As a student of both biology and religion in college, it struck me how the rise and separation of genetics from embryology, and the disparagement of the parent discipline by some of the acolytes of the new discipline, echoed the supersessionist rhetoric of Christianity as it separated from Judaism. Even more interestingly, some of the founders and critics of early genetics seemed to think so, too [12]. Morgan claimed that while geneticists kept the faith, embryologists had “run after false gods” [13]. Genetics was to replace embryology. There were many reasons for the dominance of genetics during the 20th century, not the least of which were the destruction of the Continental European laboratories during the two World Wars and the fear of mutations caused by the detonation and testing of atomic bombs [14,15].

As English replaced German as the language of science, so genetics replaced physiology and development, including theories of development as the motor of evolution. Early evolutionary theories, such as those of Robert Chambers and Charles Darwin’s grandfather, Erasmus, were based on embryonic development, especially developmental morphology. Chamber’s sensational and widely selling *Vestiges of the Natural History of Creation* was the first book “to link a developmental view of the world with evolution” [16]. Using von Baer’s principles of development, Chambers [17] argued that animal biodiversity was caused by alterations of embryonic development. In fact, Darwin explicitly viewed plant biodiversity as being predicated by alterations of floral development [18]. He also noted that natural selection could not produce the variations that provided the raw material for natural selection [18–20]. When Darwin’s theory was published, his contemporaries assumed that development was the motor that generated the variations that could be selected. Darwin’s continental champion, Ernst Haeckel [21] made embryology the key to phylogeny, and Darwin’s aggressive British champion, Thomas Huxley, wrote to Darwin that the differences between species could be traced back to the modifications of development. Evolutionary biologists such as Huxley and Herbert Spencer were greatly influenced by embryologist K. E. von Baer’s theories of development [22,23]. Indeed, when Huxley was writing [24], the word “evolution” could be used for both the individual or the species.

That view shifted with the advent of genetics. Rather than viewing evolutionary biology as the study of macroevolution, Morgan [11,25,26] would claim that only the study of intraspecies genetics was the “scientific” approach to evolution and that anything else (embryology and paleontology, to be sure) was “unscientific” and “philosophical.” He and his students carried the day (except in Russia, which viewed genetics as bourgeois metaphysics and retained an embryological view of evolution). In 1959, the centenary of Darwin’s volume, the Genetics Society of America undertook a public relations campaign to promulgate the message that Darwinism was correct because it could be fully explained by genetics. This was important because it would quiet both the Creationists in America and those scientists who favored Lysenko, the leader of Soviet biology, who embraced a Lamarckian theory of acquired heritability [27]. Embryology had given rise to the first mechanistic theories of evolution, only to be usurped by its rebellious child, genetics. Evolutionary developmental biology is now emphasizing that the emergence of new phenotypes occurs during embryonic development, and that developmental regulatory genes are crucial for evolution. Evolutionary biology cannot explain evolution by population genetics, alone. Knowledge of development is critical in explaining the origins of species. And this, as I explained to the graduate student, is the return of the rightful sovereign.

Neurobiology similarly has an embryological pedigree, and in the early 1900s, one of its biggest concerns was whether the axon was really a cellular process that extended meters in the body. Ross Granville Harrison’s inaugural tissue culture experiments [28] solved the problem by showing that the developing frog soma extended an enormous neurite. He and others also demonstrated signaling’s role in completing synapse formation and mediating the embryonic cues that guide axons from the original cell to its destined target. Through these studies of neural development, Harrison solved the problem that had so perplexed Ramón y Cajal and others who had sought to explain the patterns of neural connections in the adult body [29,30].

In 1859, the same year Darwin’s *On the Origin of Species* was published, Rudolf Virchow’s classic volume, *Cellular Pathology*, drew on embryology to explain pathology. Cancers, he argued, should be studied as errors of development because tumors appeared “by the same law, which regulated embryonic development” [31]. In the 1920s and 1930s, those embryonic laws were beginning to be explained by morphogenetic fields, and as early as 1935, C. H. Waddington [32] claimed that cancers could be studied as derangements of morphological fields established in the embryo. Tumors were seen as recapitulations of or truncated stages of normal development, and oncology emerged from the work of developmental biologists studying how misregulation leads to aberrant growth. During the mid-to-late 20th century, there was a fascinating reciprocal interaction between the two disciplines, as developmental biology provided mechanisms for cancer growth and cancer biology became a niche in which developmental biology could be nourished (i.e., get funding) [33,34]. Scientists such as T. Boveri, G. B. Pierce, and R. Auerbach used embryological means to study tumors and used tumors to study embryology. The breakthroughs in cloning were done on cancer grants to study gene regulation [35].

Yet, genetics soon assumed dominance over the field of cancer research just as it had with evolutionary biology (whose paradigms cancer biologists often propose for their own field). The founding document of the genetic (somatic mutation) theory of cancer appears to be that of Boveri [36]. Boveri was very much a cytologist and an embryologist, and he related the anomalies of cancer to those developmental anomalies caused by polyspermy and by chromosome elimination during nematode development, noting that such chromosomal rearrangements might be the cause of cancer. (Indeed, as Wunderlich [37] has shown, Boveri seems to be totally unaware of Morgan’s data for genes and did not use the term “mutation” at all. This was a later addition, probably by Morgan). The somatic mutation theory (SMT) still holds sway, claiming that cancer was due to mutations in the premalignant cell. Reviewing the embryological mechanisms of cancer, Cofre and Abdelhay [38] have recently written that “embryologists have expressed timidly” the idea that cancer can be seen as alterations of normal development and have met “with little success in leveraging the discussion that cancer could involve a set of conventional interactions used to build the embryo during morphogenesis.” However, I cannot view Barry Pierce’s [39] article “Carcinoma is to embryology as mutation is to genetics” as timid (it demands changes in the college curriculum), nor do Carlos Sonnenschein and Ana Soto, the founders of the Tissue Organizational Field Theory [40,41], hide the light of developmental cancer origins under a bushel. This failure to gain traction for a developmental approach to cancer is more likely due to the inability of the target to respond. But things may be changing. The basis for the allele-oriented SMT has recently been questioned [39–41], and the relevance of embryonic fields to cancer has been re-established [38–44]. Alterations in paracrine factor signaling in both the target and producer cells have been seen to initiate cancer formation, and embryonic processes such as epithelial-mesenchymal transformation are now seen as critical in metastasis. It is without question, though, that developmental biology helped establish oncology and has continued to help mold it. The rightful sovereign returns.

Having generated cell biology, immunology, genetics, neurobiology, and oncology, developmental biology still seems to be budding off new disciplines. Evolutionary developmental biology sees evolution as Huxley did, as changes in development (rather than changes in allele frequency) and focuses on the arrival of the fittest. Ecological developmental biology sees the environment as having instructive as well as permissive agency in normal development. Systems biology, which began with embryologically oriented philosophers such as Woodger and von Bertalanffy [45–47], attempts to fuse developmental biology, ecology, and physiology into an integrative science of becoming.

And other new disciplines are struggling to form an identity separate from their developmental parent discipline. Stem cell biology has its own meetings, its own journals, and its own professional societies, different from those of developmental biology. When Irving Weismann, one of the founders of the International Society for Stem Cell Research, became president of that organization, he threw down the gauntlet to developmental biology, saying [48],

“We are a field, a discipline, and an entire branch of science that brings new ideas, experiments, concepts, and medical translation. Like anything new, we are a threat to the established order, and at every kind of educational and research institution, to thrive, we must be recognized as entities, not as divisions of old entities.”

But it is not yet a truly independent field, as it has yet to propose anything different from developmental biology. All the articles in *Stem Cell Reports* are papers that would find a home in journals of developmental biology. At the moment, stem cell biology is a political, rather than an intellectual, bud from developmental biology, and it is performing important services in creating science-based educational accessibility and political guidelines, which the developmental biology societies have not done. Whether it becomes more than a medical aspect of developmental biology remains to be seen.

There are three main messages of this essay. The first is that developmental biology is not a confined, specified discipline—such as genetics, cell biology, immunology, oncology, neurobiology, and so forth. Developmental biology is not confined to any level of organization (in that genes, cells, tissues, organs, organisms, and ecosystems can each be studied developmentally). It can be studied in any species, organ system, or biome. Developmental biology remains pluripotent. The descendants of developmental biology—cell biology, genetics, immunology, neurobiology—are more differentiated and their potency much more restricted. They have boundaries. Surely, developmental biology has its own set of questions, perhaps the best questions of any science—How does the brain form? How do the bones of the arms become different from the bones of the legs, and why can’t we regenerate them like salamanders do? How do testes usually originate in people with a Y chromosome and ovaries in people with two X chromosomes? (And these are only a few of the questions in humans)—and it regenerates itself constantly as new techniques and hypotheses become available. Indeed, developmental biology has been called an “erotetic science,” differing from most other sciences in that it is driven by questions, not theories [49]. Thus, developmental biology is a stem cell discipline, one that regenerates itself while permitting some of its descendants to develop into their own fields.

The second message is that developmental biology remains a vital generative science. The induced pluripotential stem cells (iPSCs) are derived from the principles and discoveries of developmental biologists, as are the human beta-pancreatic cells now in clinical trials. The neural embryoids derived from such cells are now being used to study the mechanisms by which the Zika virus causes microcephaly. The 3D structure of chromatin and its remodeling during early mammalian development is becoming known, as are the mechanisms of X-chromosome inactivation. Developmental biology is also being expanded by identifying the interactions of the zygote-derived cells with those of symbiotic microbes to form organ gut, capillary, and immune cells. We are discovering how the turtle gets its shell and how the butterfly wing develops structural colors. We are in a new golden age of developmental biology.

The third message of this essay is that in the 21st century, many of the disciplines that had come from developmental biology are returning to a developmental framework, even if they don’t call it “developmental biology.” This is probably because developmental biology has always been a science about relationships in which context is critical [50], and the biology of the 21st century is focusing on relations, process, and context, rather than on entities. Thus, modern biology has come to the place where developmental biology has always been residing, a place of context-dependent interactions. Being relatively undifferentiated does not mean that developmental biology is immature [47,49–51]. Indeed, it is a science that was initiated with Aristotle and is now at the forefront of contemporary theories and methods. We can expect that even if developmental biology is not mentioned by name, the principles of developmental biology are becoming a framework integrating disciplines across biology.

## Abbreviations

iPSC: induced pluripotential stem cell
SMT: somatic mutation theory
